# Capacity value of energy storage considering control strategies

**DOI:** 10.1371/journal.pone.0178466

**Published:** 2017-05-30

**Authors:** Nian Shi, Yi Luo

**Affiliations:** State Key Laboratory of Advanced Electromagnetic Engineering and Technology, Huazhong University of Science and Technology, Wuhan, Hubei, China; Chongqing University, CHINA

## Abstract

In power systems, energy storage effectively improves the reliability of the system and smooths out the fluctuations of intermittent energy. However, the installed capacity value of energy storage cannot effectively measure the contribution of energy storage to the generator adequacy of power systems. To achieve a variety of purposes, several control strategies may be utilized in energy storage systems. The purpose of this paper is to study the influence of different energy storage control strategies on the generation adequacy. This paper presents the capacity value of energy storage to quantitatively estimate the contribution of energy storage on the generation adequacy. Four different control strategies are considered in the experimental method to study the capacity value of energy storage. Finally, the analysis of the influence factors on the capacity value under different control strategies is given.

## Introduction

With the strong demand to protect the environment and traditional energy sources becoming increasingly tense, renewable energies are considered as clean energy to minimize environmental impacts. The penetration of renewable energy into the power system is quickly increasing. However, with the integration of renewable energy, the intermittent characteristic adversely affects the reliability of power systems. Energy storage is a promising technology to smooth output power fluctuations, and it effectively improves the generation adequacy of the power system integrated with renewable energy.

Current work involving generator capacity is mainly centered on such renewable energies as wind power [1–4], solar energy [5] and tide power [6–8]. Capacity value is usually adopted to assess the contribution of renewable energy to the generation adequacy [9–11]. It is first proposed to measure the contribution of wind power to the generation adequacy [9, 12–14]. With the wide use of solar power, the capacity value of the photovoltaics [15] and concentrated solar power [16–19] are also investigated.

Existing studies consider energy storage devices as a reserve energy source to smooth the output power of renewable energy [20–24] and the effects on system reliability is ignorable. However, energy storage devices are also utilized for load shifting and peak smoothing, which effectively contribute to system reliability. When the load demand cannot be satisfied by the generators in the power system, it may be supplied by the energy stored in energy storage devices. The available capacity supplied by energy storage must be determined. With regard to the capacity of energy storage, the contribution of the capacity of energy storage to the power system is investigated integrated with wind power [20, 25–28]. Reference [25] presents a dynamic planning method to study the capacity value of energy storage, but the effects of the properties of the device and the operational strategies on the available capacity of energy storage are not mentioned. Therefore, it is necessary to establish a separate energy storage evaluation model to quantitatively estimate the contribution of energy storage to system reliability. The method is capable of providing a basis for the planning and construction of power systems.

In terms of the contribution of energy storage to the reliability of power systems, in existing studies, the analysis of the overall reliability of energy storage and renewable energy is given after accounting for energy storage. Reliability is evaluated in the power system with energy storage and wind turbines. The contribution of energy storage and wind turbines to the generator adequacy is assessed [29–33]. The energy storage is used as the reserve source of the renewable energy, and the quantitatively analysis of the capacity contribution of energy storage has been analyzed quantitatively [34]. Energy storage such as batteries [35–37] and ultra capacitors [38–41] has its own characteristics. The output power of energy storage is restricted by its own inherent attributes and energy management [42]. Otherwise, energy storage could be operated by the wind farm owner or the power system operator; the different operation strategies [43–47] largely affect the contribution of energy storage to system reliability.

Energy storage systems’ available capacity is influenced by several factors such as wind power penetration, the variability of wind power and load profile and the correlation between wind power production and load demand. The control strategy is the dominant factor influencing the available capacity of energy storage systems. However, in most existing studies there has been no comprehensive framework to evaluate the effects of the properties of the device and the operation strategies on the available capacity of the energy storage system. Therefore, it is necessary to establish a separate energy storage evaluation model to quantitatively estimate the contribution of energy storage to system reliability. Intent on revealing the relationship between available capacity and control strategies of energy storage systems, this paper presents possible control strategies in available capacity assessment models of energy storage systems containing wind power. The method can provide a basis for the planning and construction of power systems.

The key contributions of the work are as follows:

1. A metrics are used to quantitatively weigh the contribution of energy storage and provide a basis for the system planners. The renewable energy-oriented available capacity metrics are extended to access the energy storage system generator adequacy contribution with different control strategies;
2. Give energy storage capacity values with different control strategies and their sensitivity to different influences factors, such as maximum energy of energy storage, maximum discharge power of energy storage, peak load of the power system, installed capacity of wind turbines, and correlation with the capacity value of energy storage;
3. Present the control strategy oriented models of energy storage generator adequacy contribution; modeling of energy storage considering several possible operation strategies and its own characteristics is established for reliability evaluation. The effect of four different operational strategies and their parameters on the capacity value of energy storage is investigated. The proposed empirical model considers the influence factors affecting the estimation of the energy storage system capacity value. This paper also presents the principle of decreasing marginal influence factors of energy storage systems.

The definition of the capacity value of energy storage is introduced. Then, four different control strategies of energy storage are presented and the calculation of the capacity value of energy storage based on the Monte Carlo Method is given. Finally, a case study illustrates the efficiency of the model. The effects of the rated power of wind turbines, the peak load and the variables of energy storage are also analyzed in Section 5.

## Materials and methods

Generation adequacy is utilized to measure the ability of the generator devices to satisfy the load demand in power systems. The integration of intermittent energy challenges the reliability of power systems. By peak load shifting, energy storage systems effectively improve the reliability of power systems integrated with renewable energy. To quantitatively assess the available capacity of energy storage, a reliability index is established to measure the reliability of a power system; the contribution of energy storage devices to power generation is quantitatively assessed.

### Reliability index

To measure the contribution of energy storage devices to the reliability of power systems, an index for system reliability is established in this section. Using the reliability index, the capacity value of energy storage with different control strategies can be quantitatively obtained.

The reliability index of a power system at hour i of the simulation time T can be calculated as follows.

$$ReliabilityIndex_{i}=P\left(G_{load,i}>G_{tot,i}\right)=P\left(G_{load,i}>\left(G_{RE,i}+G_{ES,i}+\sum_{g=1}^{k}G_{g,i}\right)\right)$$ (1)

where *ReliabilityIndex*_*i*_ is the reliability index of the system at hour *i*. Because the reliability index is a curtailment index, the index is related to the curtailment of power system at hour *i*, (the frequency, probability and capacity of the curtailment); *G*_*load*,*i*_ is the load demand at hour *i*; *G*_*tot*,*i*_ is the total available capacity of the power system at hour *i*. In addition, the available capacity includes the available capacity of fuel generators *G*_*g*,*i*_, renewable energy generators *G*_*RE*,*i*_ and energy storage system *G*_*ES*,*i*_.

After the state of the system and load in a unit time are obtained, the reliability index of the system at corresponding unit time can be calculated by Eq (1). By accumulating the reliability indexes of all the sampling intervals in the sampling period, we can determine the reliability index of the system. Therefore, the reliability index of a power system in the simulation period is expressed as:

$$ReliabilityIndex=\sum_{i=1}^{n}ReliabilityIndex_{i}$$ (2)

where *n* is the total number of hours in the total simulation period T.

### Capacity value of the energy storage system

The contribution of the energy storage devices to system reliability can be evaluated by analysis of the impact of the installed energy storage on system reliability. According to the reliability index and the installed capacity of the system, the corresponding function is established, and from Eq (1), the corresponding relationship is expressed as follows.

$$ReliabilityIndex=f\left(G_{load,i},G_{g,i},G_{RE,i},G_{ES,i}\right)$$ (3)

where *ReliabilityIndex* is the reliability index of the power system in the sample period; G_*g*,*i*_ is the output power of fuel generators at hour i; G_*RE*,*i*_ is the output power of renewable energy generators at hour i, and G_*ES*,*i*_ is the output power of the energy storage system at hour i.

Then, derived from Eq (1), the reliability index of a power system with energy storage devices is expressed as follows:

$$\begin{array}{l} ReliabilityIndex_{ES}=\sum_{i=1}^{n}P\left(G_{ES,i}<\left(G_{g,i}+G_{RE,i}-G_{load,i}\right)*\Delta t\right)\\ =\sum_{i=1}^{n}P\left(\left(E_{ES,i-1}+\Delta t*G_{ES,i-1}\right)<\left(G_{ES,i}<\left(G_{g,i}+G_{RE,i}-G_{load,i}\right)*\Delta t\right)\right) \end{array}$$ (4)

where *E*_*ES*,*i-*_1 is the energy stored in energy storage at hour i−1, G_*ES*,*i-*_1 is the output power of energy storage at hour i, and t is the sampling interval at i.

A unit generator is installed in the power system instead of the energy storage unit. The reliability index of the power system integrated with the unit generator is expressed as follows:

$$ReliabilityIndex_{CU}=\sum_{i=1}^{n}P\left(G_{U}*\Delta t<\left(G_{g,i}+G_{RE,i}-G_{load,i}\right)*t_{i}\right)=F\left(Energy_{CU}\right)$$ (5)

where *G*_*U*_ is the capacity value of the added unit generator.

Then, the capacity value of G_*U*_ is adjusted, until:

$$F\left(Energy_{ES}\right)=F\left(Energy_{CU}\right)$$ (6)

The capacity value of G_*U*_ that fulfills Eq (6) is defined as the capacity value of the energy storage system. It is described as

$$G_{CVES}=G_{U}$$ (7)

where *G*_*CVES*_ is the capacity value of the energy storage system.

## Wind/Battery energy storage system model

### Wind power model

The output power of wind turbines is related to wind speed and the parameters of the wind turbine. The output power of a wind turbine can be divided into four ranges according to the related wind speed [48]. The output power of a single wind turbine can be calculated as:

$$P_{Wind}=\{\begin{array}{ll} 0, & 0\leq v<C_{in}\\ P_{r}*\left(C_{1}+C_{2}*v+C_{3}*v^{2}\right), & C_{in}\leq v<C_{rate}\\ P_{r},\; & C_{rate}\leq v<C_{out}\\ 0, & C_{out}\leq v \end{array}$$ (8)

where *P*_*r*_ is the rated power of the wind turbine; C_*1*_, C_*2*_ and C_*3*_ are the parameters related to wind speed and estimated by historical data. C_*in*_, C_*rate*_ and C_*out*_ are the cut-in, rated and cut-out wind speed, respectively.

Historical wind speed data covering a sufficient time span is used for the wind speed characteristic analysis [48–50]. Yearly historical chronological wind speed data is utilized to describe the wind power model. In this study, 10-year historical wind speed data at Prince Albert in Canada is adopted.

### Energy storage system model

The characteristics of intermittent energy, such as wind and photovoltaic energy, considerably affect the reliability of a power system. Energy storage is capable of effectively providing reserve energy; it is widely utilized for load shifting and peak smoothing. To quantitatively measure the capacity value of energy storage, the model of energy storage considering operation strategy is given in this part.

The modeling of energy storage includes operation strategies, available energy stored in the devices and maximum charging and discharging power. The contribution of energy storage to adequate power supply depends on the output power of the energy storage (restricted by the maximum permissible charging and discharging power), the surplus energy stored in energy storage, the maximum and minimum energy limits, and the operation strategy [29]. Therefore, the power output of the energy storage system can be expressed by a function as:

$$G_{ES_{output}}=f\left(P_{cha},P_{discha},E_{bat},P_{Ava}\right)$$ (9)

where *G*_*ESoutput*_ is the power output of the energy storage system, *P*_*cha*_ and *P*_*discha*_ are the charging and discharging power of energy storage, respectively, *E*_*bat*_ is the surplus energy stored in energy storage and *P*_*Ava*_ is the available power of energy storage.

#### Charging and discharging

To clearly understand the capacity value of energy storage, charging and discharging functions are described in this section. The operation strategies for energy storage are stated as follows.

The charging power of the energy storage at hour *i* is:

$$P_{cha,i}=\{\begin{array}{ll} P_{EScha\_Max}, & P_{Sup,i}\geq P_{EScha\_Max}\\ P_{Sup,i}, & P_{EScha\_Max}>P_{Sup,i}\geq 0 \end{array}$$ (10)

The discharging power of the energy storage at hour *i* is:

$$P_{discha,i}=\{\begin{array}{ll} P_{ESdischa\_Max}, & -P_{Sup,i}\geq P_{ESdischa\_Max}\\ -P_{Sup,i}, & P_{ESdischa\_Max}>-P_{Sup,i}\geq 0 \end{array}$$ (11)

where *P*_*EScha_Max*_ and *P*_*ESdischa_Max*_ are the maximum charging and discharging power of the energy storage system, respectively. They are the constraints on charging and discharging power. *P*_*Sup*,*i*_ is the surplus power of the power system with energy storage devices.

The model of the energy storage system is also related to operation strategy. The energy storage system can be operated by the wind farm owner or the power system operator [20]. Therefore, the possible operation strategies are stated as Strategy 1 to Strategy 4.

#### Strategy 1

To improve the supply reliability of a power system and reduce load shedding, Strategy 1 is presented. In this strategy, the power output of wind turbines and fuel generators are all used to charge energy storage.

1. When the sum of the power output of the wind turbines and fuel generators is more than the load demands, the surplus power will charge energy storage;
2. If the sum of the output power of the wind turbines and fuel generators cannot satisfy the load demand, then the energy storage system will release energy to support the demand.

Thus, *P*_*Sup*,*i*_ in the charging and discharging equation is calculated as

$$P_{Sup,i}=G_{RE,i}+\sum_{g=1}^{k}G_{g,i}-G_{load,i}$$ (12)

In Eq (12), if *P*_*sup*,*i*_>0, energy storage stores the energy and works at charging status; and if *P*_*sup*,*i*_<0, energy storage discharges energy.

#### Strategy 2

Because of constraints on wind power penetration levels in some power systems, Strategy 2 is proposed. In this strategy, the sum of the power output of wind turbines and energy storage is not supposed to exceed X% of the load demand (X% = 30% usually). Considering that the owners of the energy storage system and fuel generators are not the same business, the charging power of the energy storage system from fuel generators is not considered.

1. If the output power of the wind turbines is less than X% of the load demand and there is available power in the energy storage system, then energy storage will supply the load to maintain the total output of wind power and energy storage at X% of load demand.
2. If the output power of the wind turbines is less than X% of the load demand and the energy storage system cannot supply the load demand, then the fuel generators will satisfy the remainder of the load demand, and energy storage would not charge or discharge power.
3. If the output power of the wind turbines is more than X% of the load demand, then the surplus power will be utilized to charge the energy storage system. The remainder of the load demand is satisfied by fuel generators.

Thus, *P*_*Sup*,*i*_ is expressed as:

$$P_{Sup,i}=G_{RE,i}-X\%*G_{load,i}$$ (13)

In Eq (13), if *P*_*sup*,*i*_>0, energy storage stores the energy and works at charging status; if *P*_*sup*,*i*_<0, energy storage discharges energy.

#### Strategy 3

Strategy 3 is similar to Strategy 2. However, in this strategy, the fuel generators, wind turbines and energy storage are supervised by the same organization. Energy exchanges are allowed among them. Therefore, operation Strategy 3 is as follows.

1. When the output power of the wind turbines is less than X% of the load demand, energy storage will supply the load. The fuel generators will satisfy the remainder of the load demand.
  Thus, if *G*_*RE*,*i*_ < *X*% *G_*load*,*i*_ and *E*_*bat*_ > *E*_min_, the energy storage system discharges. The *P*_*Sup*,*i*_ in Eq (11) can be calculated by
  $$P_{Sup,i}=G_{RE,i}-X\%*G_{load,i}$$ (14)
2. If the output power of the wind turbines is less than X% of the load demand and the energy storage system cannot supply the load demand, then the fuel generators will satisfy the remainder of the load demand, and energy storage will not charge or discharge power.
  Thus, the energy storage system discharges. The *P*_*Sup*,*i*_ in Eq (11) can be calculated by
  $$P_{Sup,i}=0$$ (15)
3. When the available power of the wind turbines is greater than X% of the load demand, but the power output of the fuel generators is lower than (1-X%) of the load demand, energy storage can be used to satisfy the load demand.

If *P*_*Sup*,*i*_ < 0 and $\sum_{g=1}^{k}G_{g,i}-\left(1-X\%\right)*G_{load,i}<0$, the discharging equation of energy storage is expressed as Eq (11), and the *P*_*Sup*,*i*_ in Eq (11) can be calculated by

$$P_{Sup,i}=G_{RE,i}+\sum_{g=1}^{k}G_{g,i}-G_{load,i}$$ (16)

If *P*_*Sup*,*i*_ ≥ 0 and $\sum_{g=1}^{k}G_{g,i}-\left(1-X\%\right)*G_{load,i}<0$, the discharging equation of energy storage can be calculated by

$$P_{discha,i}=\left\{ \begin{array}{l} \sum_{g=1}^{k}G_{g,i}-\left(1-X\%\right)*G_{load,i},-P_{ESdischa\_Max}<\sum_{g=1}^{k}G_{g,i}-\left(1-X\%\right)*G_{load,i}<0\\ -P_{ESdischa\_Max},\sum_{g=1}^{k}G_{g,i}-\left(1-X\%\right)*G_{load,i}\geq -P_{ESdischa\_Max} \end{array}\right.$$ (17)

where $P_{Sup,i}=G_{RE,i}+\sum_{g=1}^{k}G_{g,i}-G_{load,i}$.

#### Strategy 4

This strategy is used to smooth the wind power. In the sample period, we calculate the average power of the wind turbines. The operation strategy is detailed as follows.

1. When the power output of the wind turbines is greater than the average power of the wind turbines, the surplus power will be stored in energy storage.
2. When the power output is smaller than the average power, the surplus power of load demand will be satisfied by energy storage.

Thus, *P*_*Sup*,*i*_ in Eqs (10) and (11) is expressed as:

$$P_{Sup,i}=G_{RE,i}-G_{Wind\_Ava}$$ (18)

where *G*_*Wind_Ava*_ is the average power output of the wind turbines in the sampling period.

#### Energy stored in the energy storage

The energy stored in energy storage at the sampling interval *i* is related to the energy at the previous interval. The energy stored in energy storage at the sampling interval *i* + 1 is calculated as

$$E_{ES,i+1}=\left\{ \begin{array}{l} E_{ES,i}+T*P_{ES_{output}},E_{\text{min}}\leq E_{ES,i}+T^{-1}*P_{ES_{output}}\leq E_{\text{max}}\\ E_{\text{min}},E_{ES,i}+T^{-1}*P_{ES_{output}}\leq E_{\text{min}}\\ E_{\text{max}},E_{\text{max}}\leq E_{ES,i}+T^{-1}*P_{ES_{output}} \end{array}\right.$$ (19)

*E*_*max*_ and *E*_*min*_ are the maximum and minimum energy stored in energy storage devices. T represents the sampling interval. Compared to the sampling interval, the response time of battery energy storage is very small. The response time is negligible in this model [50].

Based on the four operation strategies, the PI controller is utilized to allow output power of the energy storage system to respond more rapidly to the available capacity of the power system and the variation of load. The reference value of the active power of the energy storage system is given by the PI controller. The governing equations are as follows.

$$P_{ES\_ref}=(k_{p}+\frac{k_{i}}{s})(G_{RE,i}-G_{ref})$$ (20)

where *G*_*ref*_ is the reference value of the active power of the energy storage system under different operation strategies. For the reference values, *k*_*p*_ and *k*_*i*_ are the scale coefficient and integral coefficient of the PI controller. *P*_*ES_ref*_ is the reference value of the output power of the energy storage system. When *P*_*ES_ref*_ >0, the energy storage devices are discharged; when *P*_*ES_ref*_ <0, they are charged.

## Operation principle

**Fig 1:**
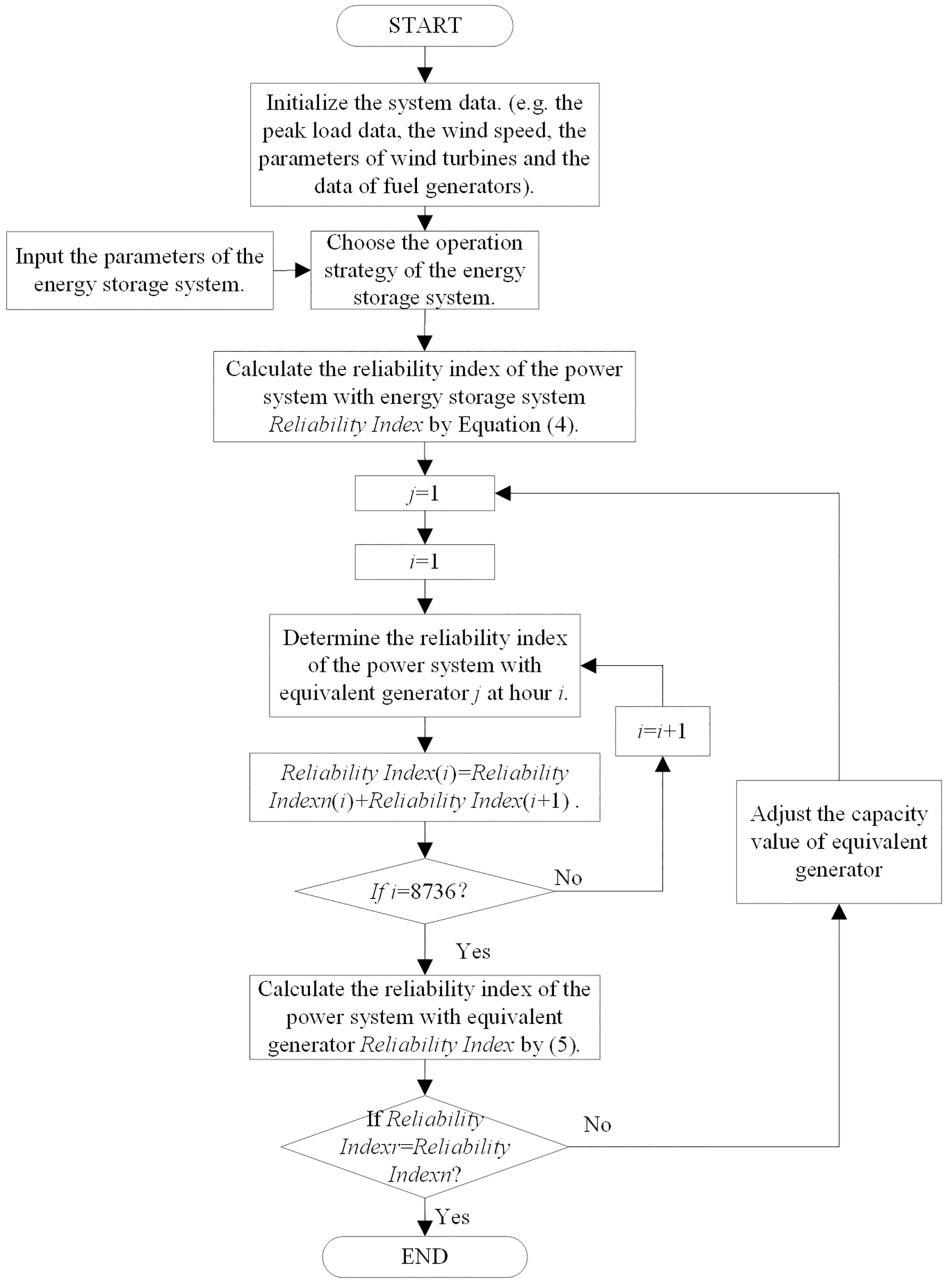
The flowchart of the capacity value of energy storage considering strategy.

A sequential Monte Carlo Method is utilized for the calculation. We can obtain the working state of the fuel generators, wind turbines and load demand at the sample interval by MC sampling, according to the time sequence and obtain the working state of the energy storage system. The flowchart of the calculation of the capacity value of energy storage considering control strategies is shown in Fig 1. The calculation process is detailed as follows:

1. Initialize the data of the original power system data, such as the wind turbine model, the energy storage model and related parameters, and the load demand, the wind speed and the fuel generators.
2. Confirm the control strategy of the energy storage system and wind turbines from the four control strategies.
3. Obtain the output power of fuel generators by the MC method, the output power of wind turbines by Eq (8) and the load demand of each sampling period. Then, calculate the output power of energy storage by Eqs (10–18).
4. Calculate and save the reliability index of the power system with energy storage in the sampling time period using Eq (4). If the available capacity cannot satisfy the load demand at hour *i*, then the Reliability Index is accumulated. Otherwise, the value of the Reliability Index remains the same.
5. Replace the energy storage system by the unit generator, and calculate the reliability index of the power system with the equivalent generator using Eq (5).
6. If the reliability index of the power system with the equivalent generator is not equal to the reliability index of the power system with energy storage, then adjust the value of the equivalent capacity *G*_*U*_.
7. Calculate the reliability index of the power system with equivalent capacity.
8. Determine whether Eq (6) is met. If Eq (6) is met, go to Step 9). In addition, if not, go to Step 6).
9. Store the value of the equivalent capacity; the equivalent capacity is defined as the capacity value of energy storage.

## Results and discussion

The assessment of the capacity value of an energy storage system under different operational strategies is examined by application of the RBTS [51]. The key factors that affect reliability will also affect the capacity value of energy storage. The reliability index with four operational strategies of energy storage is analyzed in terms of the effect of impact factors on the reliability of the power system. They are the yearly peak load, the maximum permissible energy stored in storage power and the installed capacity of wind turbines. The sensitivity analysis of these factors is investigated in the following case studies.

In this study, fuel generators, wind turbines and energy storage devices are installed in the system. There are three 100 kW fuel generators (FOR = 0.05) and one 50 kW generator (FOR = 0.02) in the system. The wind speed is the same as Prince Albert in Canada, from the years 2001 to 2012 [52]. The cut-in, rated and cut-out speeds are 3.75, 12 and 23.2 m/s, respectively. In addition, the values of C1, C2 and C3 are 0.1203, -0.08 and 0.0128, respectively. The rated power of the wind turbines is 800 kW. A sequential load model of IEEE-RTS [53] is applied in this test. For the energy storage system, P_*chmax*_ = 800 kW, P_*dchmax*_ = 250 kW and E_*max*_ = 10000 kWh.

### Comparison of the four strategies

In this part, the comparison of the capacity values of the four strategies is analyzed. As Strategy 2 and Strategy 3 are similar, the capacity values with the same dispatch restriction are close. The comparison of the capacity values of Strategy 2 and Strategy 3 is analyzed. Table 1 demonstrates the changing trend of the capacity value of energy storage with different X% of the load demand. When the wind energy dispatch restriction changes from 25% to 55% of the load demand, the changing trend of the capacity values of energy storage is shown in Table 1. Because energy storage can be used when the output power of the fuel generator is lower than (1-X %) of the load demand in Strategy 3, the capacity value of energy storage of Strategy 3 is slightly higher than Strategy 2. Therefore, the energy storage is used more fully by Strategy 3.

**Table 1:** Comparison of Strategy 2 and Strategy 3 of capacity value of the energy storage against X% of peak load.

	Strategy 2	Strategy 3
X% = 25%	234.7	236
X% = 30%	234.7	236
X% = 35%	234.7	236
X% = 40%	227.6	229
X% = 45%	217.7	219
X% = 50%	196.2	198
X% = 55%	173.7	175

As shown in Table 1, when the wind turbines and energy storage system are charged with 35% to 25% of the load demand, the wind turbines and energy storage are used fully to satisfy the load demand, thus the capacity value of the energy storage system is high. The capacity value of energy storage decreases when the supplied load demand is higher than 35%. The reason is that wind power cannot satisfy the load demand, and the reliability of the power system decreases. Therefore, the energy storage is not used sufficiently, and the capacity value of energy storage also decreases.

**Fig 2:**
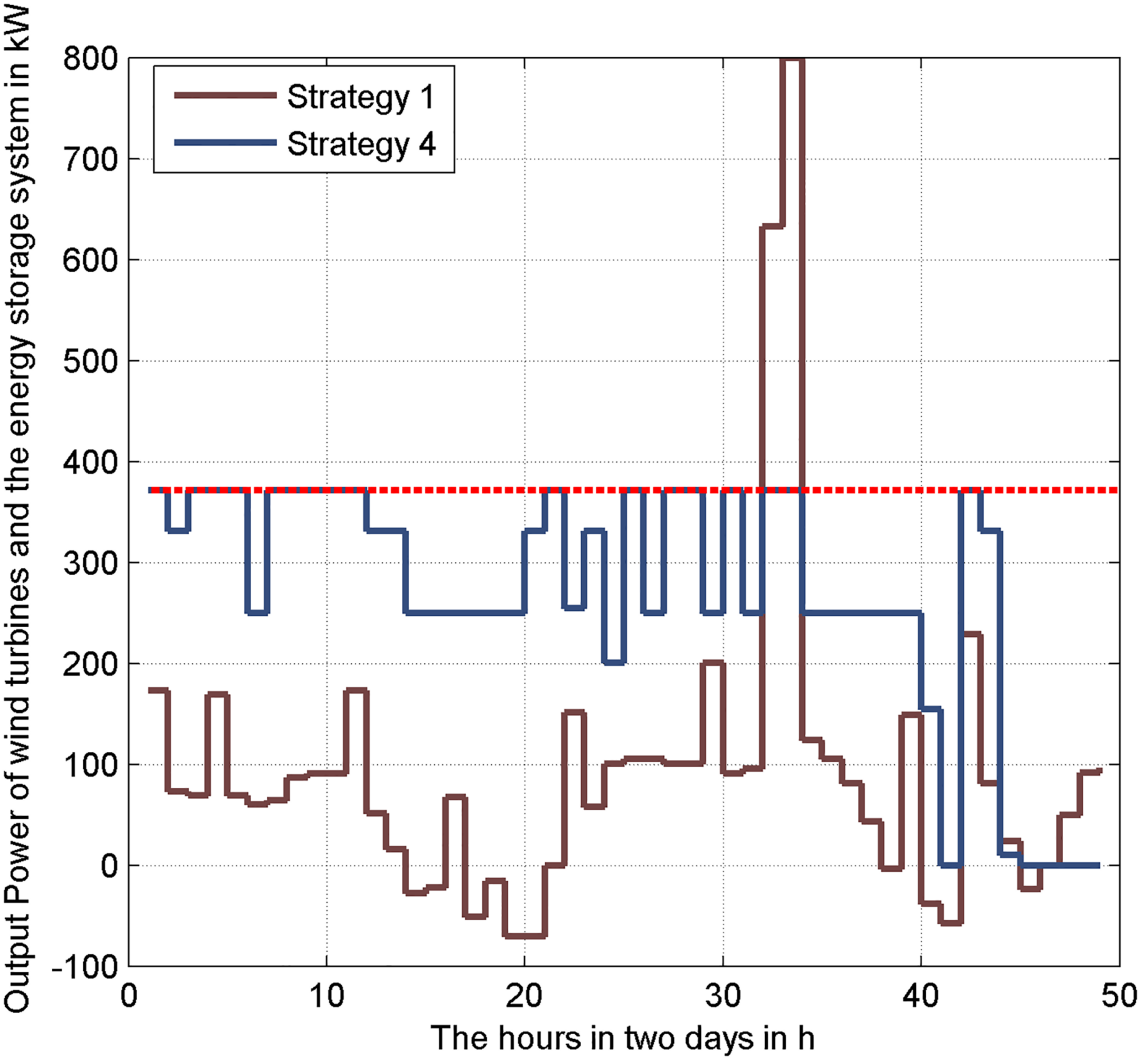
Comparison of Strategy 1 and Strategy 4 of the output power of renewable energy and the energy storage in 24 hours.

Strategy 4 is used to smooth the wind power. The output power of the wind turbines and energy storage system in 48 hours is analyzed. As shown in Fig 2, the average power of wind turbines is 371 kW. The output power of the wind turbines and energy storage system with Strategy 4 is closely to the average power of wind turbines. It has the minor wave range of power (from 0 to 371 kW). And with Strategy 1, the energy storage system is fully used to satisfy the load demand. The range of power is from -80 kW to 800 kW. Strategy 4 has a better effect on suppressing the power fluctuations than Strategy 1.

### Impact of the maximum energy of energy storage

**Fig 3:**
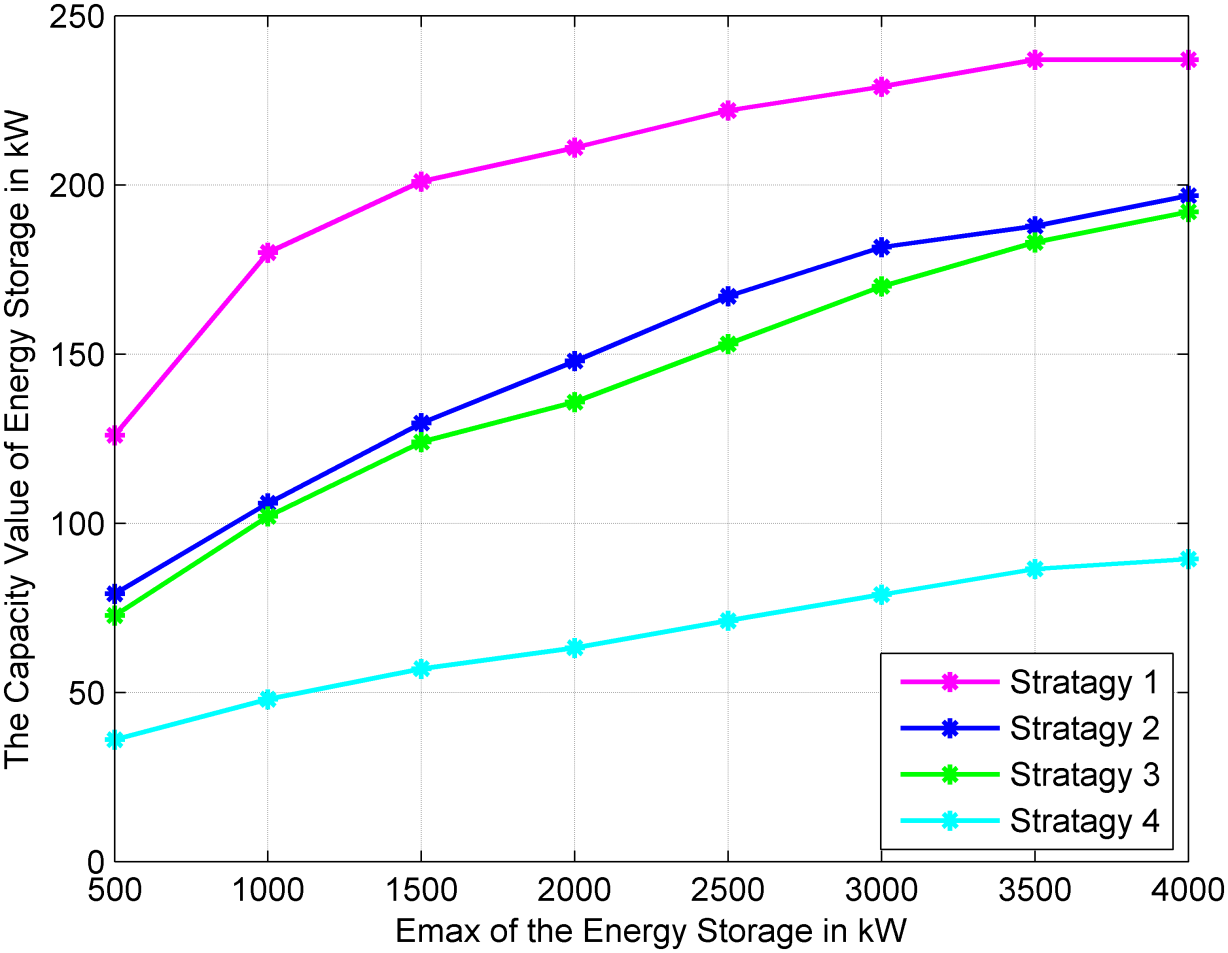
Variations of the capacity value of energy storage for different *E*_*max*_ of energy storage.

In this part, the maximum energy stored in energy storage E_*max*_ is set from 500 kW to 4000 kW. Fig 3 shows the change trend of the capacity value of energy storage with different E_*max*_ in four different strategies. In Fig 3, with the increase of E_*max*_, the capacity values of all four strategies increase with the E_*max*_ to a stable value.

As shown in Fig 3, with the increase of E_*max*_, the capacity value of energy storage in the four strategies has the same changing trend; it increases slowly until it reaches a stable value. Until the E_*max*_ increases to 3000 kWh, the capacity value reaches a constant value. In Strategy 1, because the output power of energy storage is only restricted by its own characteristic parameters, and fuel generators and wind turbines all can charge the energy storage, the capacity value of energy storage in Strategy 1 is the highest. Since the output power of energy storage is only related to the average wind power in Strategy 4, the capacity value of energy storage is lower but more stable. In Strategy 2 and Strategy 3, the capacity values are nearly the same, corresponding to the same E_*max*_. The utilization efficiency of energy storage in Strategies 2 and 3 is in the middle of Strategy 1 and 4.

### Impact of the maximum discharge power of energy storage

**Fig 4:**
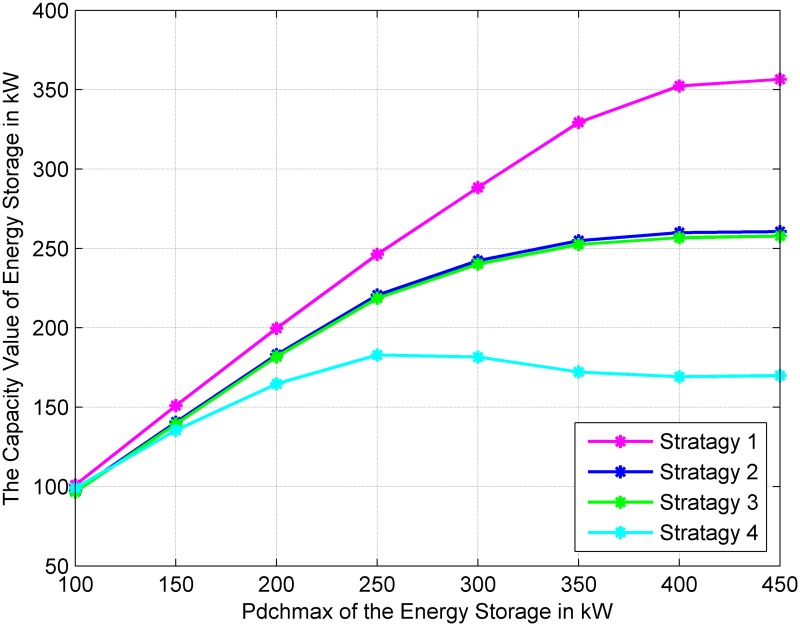
Variations of the capacity value of energy storage against different maximum discharge power values of energy storage.

With different values of the maximum discharge power P_*dchmax*_, the capacity values of energy storage have the same change trend in the four operation strategies. As Fig 4 shows, with the increase of P_*dchmax*_ of energy storage, the capacity value of energy storage increases until it reaches a stable value. This is because increasing the P_*dchmax*_, increases the output power of the energy storage system; therefore, energy storage will effectively improve the reliability of the power system. The output power of energy storage is also related to the wind power, load demand, and maximum energy stored in energy storage; therefore, when the P_*ESdischa_Max*_ increases to a certain value, the contribution of energy storage to the reliability of the power system will reach a maximum value, and the capacity value of energy storage will reach a stable value.

The curves of the four operational strategies are shown in Fig 4. The slope of the curve is more moderate in Strategy 4. For this operation strategy, the capacity value reaches the maximum value when the P_*ESdischa_Max*_ increases to 250 kW, and then it maintains this value. In Strategies 3 and 2, the capacity value reaches the maximum value when the P_*ESdischa_Max*_ increases to 350 kW and in Strategy 1 when the P_*ESdischa_Max*_ increases to 500 kW. In addition, the influence of the P_*ESdischa_Max*_ on the capacity value of energy storage in Strategies 2 and 3 are the same.

### Impact of the peak load of the power system

**Fig 5:**
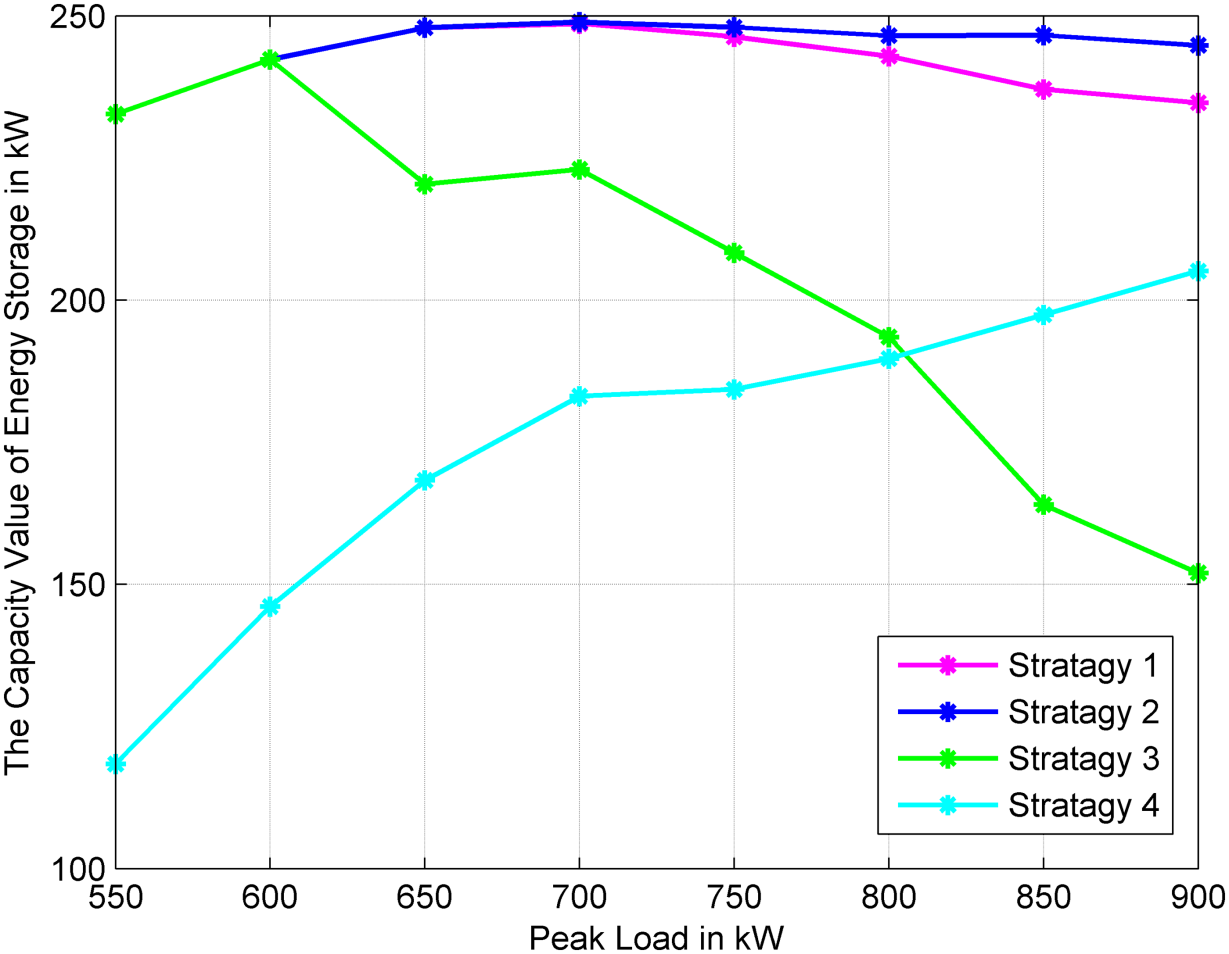
Variations of the capacity value of energy storage against different peak load.

In this part, the impact of the peak load on the capacity value of energy storage for four operational strategies is analyzed. Fig 5 demonstrates the change trend of capacity value with different yearly peak load power values.

When the yearly peak load power increases from 500 kW to 700 kW by 50 kW steps, the capacity value of energy storage is shown in Fig 5. Because the energy storage devices have been fully applied in improving system reliability, when the peak load increases from 500 kW to 700 kW, the capacity value of energy storage in Strategies 1, 2 and 3 have little increase, even a decrease in Strategy 3. However, for Strategy 4, the output power of energy storage is also related to the average power of wind turbines; therefore, with the increase in peak load, the contribution of energy storage to the system reliability also increases in Strategy 4, and the capacity value of energy storage has steady growth.

### Impact of the installed capacity of wind turbines

**Fig 6:**
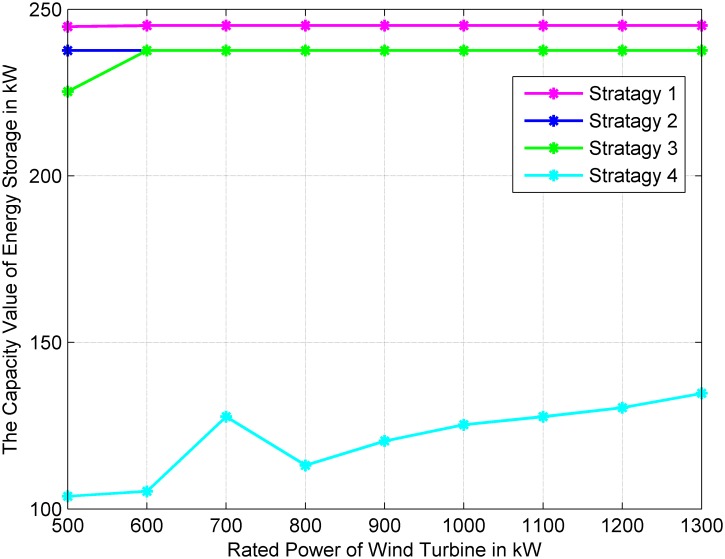
Variations of the capacity value of energy storage against different installed capacity values of wind turbines.

Fig 6 shows the capacity value of energy storage in four operation strategies when the rated output power of the installed wind turbines increases from 500 kW to 1300 kW. Compared with Strategies 1, 2 and 3, the capacity value of energy storage in Strategy 4 has the minimum value. With the increasing of the installed capacity of wind turbines, there has been a general uptrend in the capacity value of energy storage in Strategy 4. While the capacity value in other operation strategies keeps on a stable value. This is because the output power of energy storage in Strategy 4 is closely related to the wind power. Therefore, when the installed wind power is lower, the energy stored in energy storage has not been fully utilized and the capacity value is small. The main purpose of the energy storage in this strategy is to smooth the output power of the wind turbines. With the increase of the installed power of the wind turbines, the capacity value of energy storage in Strategy 4 also increases. The applied efficiency of energy storage in Strategy 4 is effectively raised.

## Conclusion

An energy storage system can effectively improve the reliability of a power system with intermittent energy. It can also be used to smooth the fluctuation of wind power. In this paper, the definition of the capacity value of energy storage is presented to quantitatively weigh the contribution of energy storage to the generation adequacy. An energy storage model considering four possible operating strategies for reliability evaluation is presented and applied to a test system. The capacity value of energy storage under four different operational strategies is analyzed and compared in terms of impact factors. The impact factors of the capacity value of energy storage are investigated in the case study.

The impact of the key factors on the capacity value of energy storage in Strategies 2 and 3 are nearly the same. The maximum discharging power of energy storage can greatly affect the reliability benefits from energy storage in Strategies 1, 2 and 3, and it will have negligible effects on the reliability benefits in Strategy 4. Comparing the four operation strategies, the capacity value in Strategy 1 is the maximum, which means that the energy storage in Strategy 1 has more capacity to improve system reliability. In Strategy 4, the capacity value of energy storage is the minimum because the mainly aim of energy storage in Strategy 4 is mainly used to smooth the fluctuant wind power and plays a minor role in improving system reliability.
